# Visually guided behavior in freely moving mice

**DOI:** 10.1186/1471-2202-14-S1-P141

**Published:** 2013-07-08

**Authors:** Balaji Sriram, Alberto Cruz-Martin, Laura DeNardo, Mohit Patel, Euiseok J Kim, Anirvan Ghosh

**Affiliations:** 1 Division of Biology, University of California, San Diego, La Jolla, USA; 2 Department of Biology, Stanford University, Stanford, USA; 3 SNL-C, Salk Institute for Biological Sciences, La Jolla, USA; 4 CNS Discovery, F. Hoffmann La Roche, Basel, Switzerland

## 

The ability of neuroscience to ascribe functions to brain regions and to different neuronal subtypes within these regions depends on our ability to identify behavioral paradigms that depend on these functions and to measure these behaviors in a quantitative fashion. Due to their inexpensive nature, extensive similarities in brain architecture, availability of genetic tools and ease of handling, rodents have recently become an important tool in the study of neuronal coding. We train common mice (*Mus musculus*) in a potentially cortex dependent, visually guided task.

**Figure 1:**
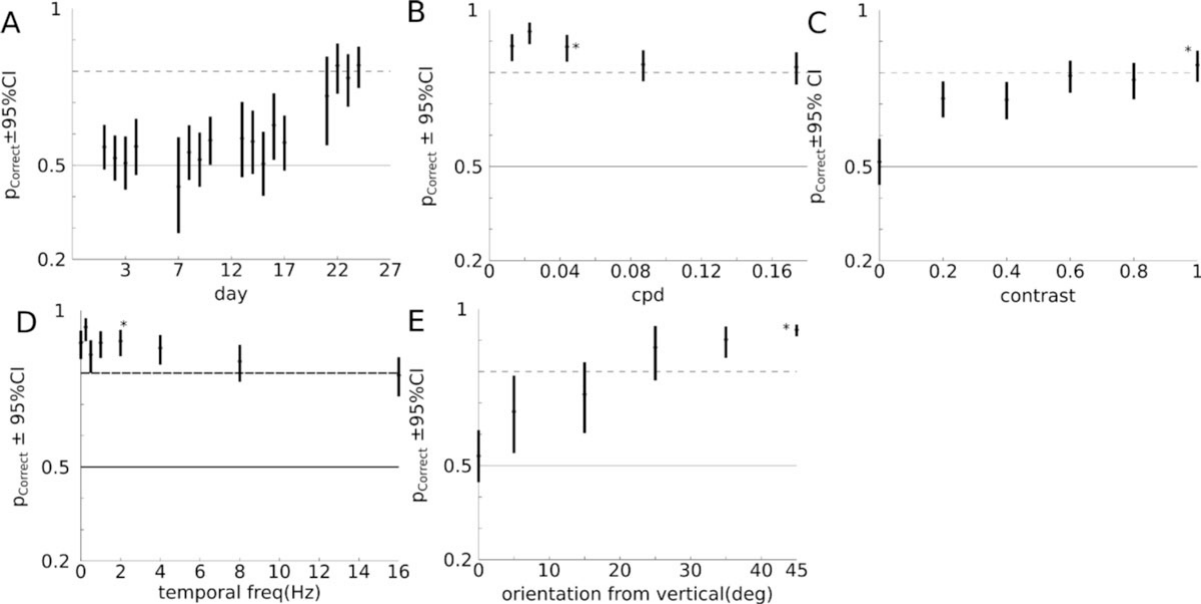
**Example psychometric curves from a typical subject**. (A) Initial training with full contrast, 0.05cpd, 2Hz gratings. After the initial training period, performance was measured with varying spatial frequency (B), contrast (C), temporal frequency (D) and orientation of grating (E). All data show binomial fits to the data with 95% confidence intervals. In B,C,D and E the condition corresponding to the original training is denoted by.'*'

Adult C57BL/6 mice were trained to perform an orientation discrimination task in a 2AFC (2 alternate forced choice) behavioral training chamber. Subjects performed trials in a semi-closed economy obtaining water through the training chamber 5-6 days a week with ad-libitum water provided for the remaining 1-2 days. During the initial training stage, stimuli were full field drifting gratings (100% contrast, ~0.05 cpd, 2 Hz) with randomly interleaved trials showing gratings oriented ±45° from the vertical. Mice performed on average ~200 trials every day. Subjects learned the task until they reached a criterion performance (80-85% correct) (Figure 1A).

Past this initial training stage, subjects were tested on a variety of stimuli where the discriminated gratings showed varying spatial frequency (Figure 1B), contrast (Figure 1C), temporal frequency (Figure 1D) and orientation (Figure 1E) from the vertical allowing us to measure precise psychometric curves on the performance of subjects.

The role of cortical (Primary visual cortex; V1) and subcortical circuits (Superior colliculus; SC) mediating this behavior will be probed through the use of lesions. We hope that such measurements will provide a basis to constrain and in the future uniquely describe models of the neocortex.

